# Structural basis of thalidomide enantiomer binding to cereblon

**DOI:** 10.1038/s41598-018-19202-7

**Published:** 2018-01-22

**Authors:** Tomoyuki Mori, Takumi Ito, Shujie Liu, Hideki Ando, Satoshi Sakamoto, Yuki Yamaguchi, Etsuko Tokunaga, Norio Shibata, Hiroshi Handa, Toshio Hakoshima

**Affiliations:** 1 Structural Biology Laboratory, Nara Institute of Science and Technology, 8916-5 Takayama, Ikoma, Nara 630-0192 Japan; 2 Department of Nanoparticle Translational Research, Tokyo Medical University, Tokyo, 160-8402 Japan; 3 PRESTO, JST, 4-1-8, Honcho, Kawaguchi, Saitama 332-0012 Japan; 4 School of Life Science and Technology, Tokyo Institute of Technology, Yokohama, 226-8501 Japan; 5 Department of Nanopharmaceutical Sciences, Nagoya Institute of Technology, Gokiso, Showa-ku, Nagoya, 466-8555 Japan

## Abstract

Thalidomide possesses two optical isomers which have been reported to exhibit different pharmacological and toxicological activities. However, the precise mechanism by which the two isomers exert their different activities remains poorly understood. Here, we present structural and biochemical studies of (*S*)- and (*R*)-enantiomers bound to the primary target of thalidomide, cereblon (CRBN). Our biochemical studies employed deuterium-substituted thalidomides to suppress optical isomer conversion, and established that the (*S*)-enantiomer exhibited ~10-fold stronger binding to CRBN and inhibition of self-ubiquitylation compared to the (*R*)-enantiomer. The crystal structures of the thalidomide-binding domain of CRBN bound to each enantiomer show that both enantiomers bind the tri-Trp pocket, although the bound form of the (*S*)-enantiomer exhibited a more relaxed glutarimide ring conformation. The (*S*)-enantiomer induced greater teratogenic effects on fins of zebrafish compared to the (*R*)-enantiomer. This study has established a mechanism by which thalidomide exerts its effects in a stereospecific manner at the atomic level.

## Introduction

More than 50 years have passed since thalidomide was first prescribed as a sedative and antiemetic to provide effective relief from morning sickness during early pregnancy. The teratogenic effects associated with its use were soon discovered along with severe birth defects such as phocomelia and amelia^1–3^. Pharmacological studies aimed at delineating the cause of thalidomide-induced teratogenicity led to the discovery of a number of unexpected pharmacological activities including anti-inflammatory, inhibition of tumor necrosis factor (TNF)-α production and anti-angiogenic effects^4–6^. Other studies demonstrated thalidomide-induced oxidative stress, which results in DNA damage or perturbation of the signaling pathways involving NF-κB or Bmp/Dkk1/Wnt^7–10^. Thalidomide and its derivatives are now widely used as potent immunomodulatory drugs (IMiDs) in the treatment of several diseases including multiple myeloma (MM) and leprosy (Hansen’s disease)^11–14^. Furthermore, thalidomide has recently been investigated in the treatment of vascular diseases^15,16^.

Thalidomide is the small synthetic compound α-phthalimido-glutarimide (IUPAC systematic name, 3-(*RS*)-2-(2,6-dioxo-3-piperidyl)isoindole-1,3-dione), which possesses one chiral centre (the C3-carbon atom of the glutarimide ring), and comprises a racemic mixture of two optical isomers, (R)- (also (+)-) and (*S*)- (also (−)-) enantiomers, currently in clinical use. The (*R)*- and (*S*)-enantiomers were once thought to be responsible for the sedative and teratogenic effects, respectively. This idea was challenged by the findings that the (*R*)-enantiomer is also teratogenic in a rabbit model^11,12^ and that interconversion of the enantiomers could occur under physiological conditions^17–19^. However, a number of reports, including some describing the use of configuration-stable thalidomide analogues, have shown that the (*S*)-enantiomer is more teratogenic and effective at inhibiting TNF-α production and angiogenesis compared to the (*R*)-enantiomer^20–24^.

Thalidomide directly binds cereblon (CRBN), which was originally reported as a cerebral protein associated with mild mental retardation^25,26^. CRBN is a highly conserved protein that forms a CRL4-type E3 ubiquitin ligase complex, CRL4^CRBN^, with Cul4A and damaged DNA binding protein 1 (DDB1), and plays a key role in limb outgrowth and expression of fibroblast growth factor Fgf8 in zebrafish and chicks^25^. Thalidomide is suggested to initiate its teratogenic effects by binding to CRBN and modulating the associated ubiquitin ligase activity^25^. Moreover, a human MM cell line with deletion of the *CRBN* gene was shown to be resistant to thalidomide derivatives, indicating that CRBN is involved in both the teratogenic and beneficial effects of thalidomide^27^. Recent crystallographic studies of human, mouse and chick CRBN have succeeded in elucidating the manner by which thalidomide and its derivatives bind the tri-Trp pocket formed by three conserved surface tryptophan residues in the thalidomide-binding domain (TBD) of CRBN^28,29^. In these studies, however, all experiments were performed using racemic compounds, hence enantiomer-specific differences in CRBN binding were not addressed. The precise manner by which these enantiomers exert their different pharmacological and toxicological effects remained unanswered. Here, we report on a series of crystallographic and biochemical studies investigating the interaction between each thalidomide enantiomer and CRBN, and provide a structural basis for the differences in CRBN binding and stereospecific effects of thalidomide on teratogenicity.

## Results

### Differences in CRBN binding affinity between (*S*)*-* and (*R*)- thalidomides assayed using deuterium-substituted enantiomers

**Figure 1:**
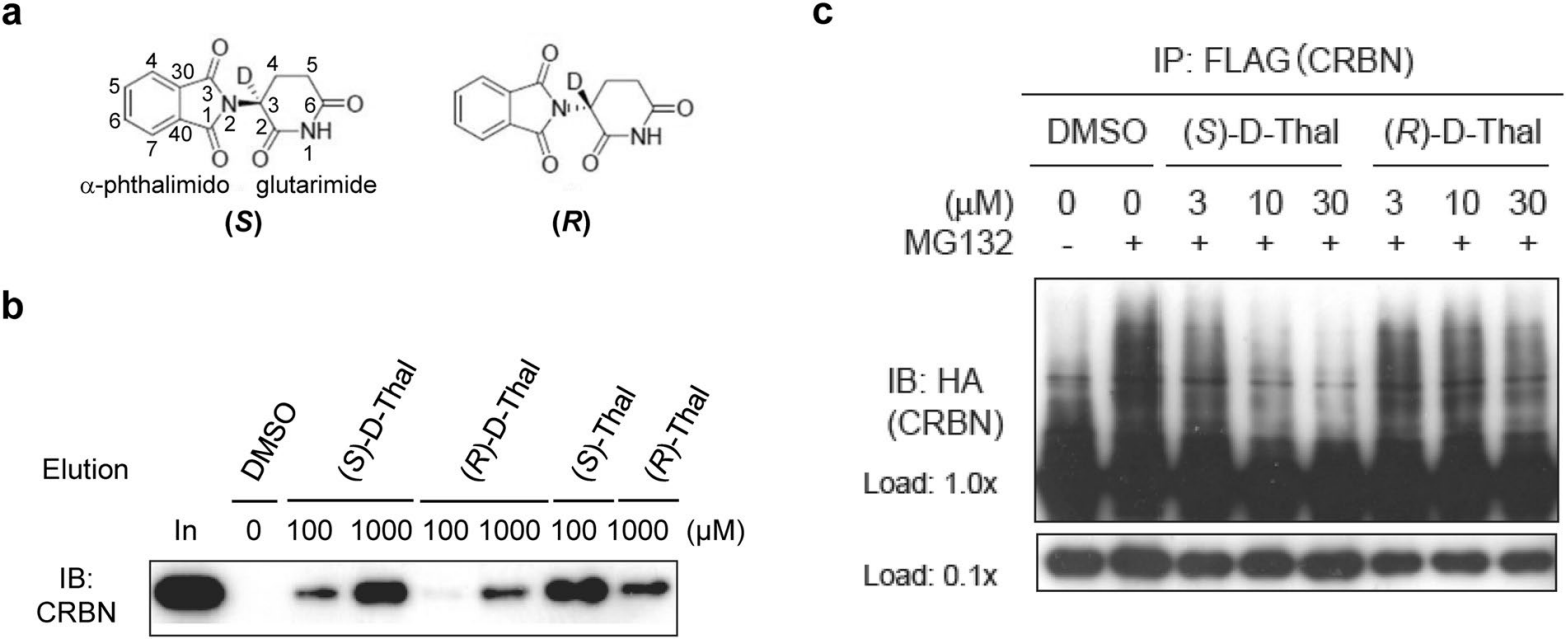
Binding assays of deuterium-substituted (*S*)- and (*R*)-thalidomides with human CRBN TBD. (**a**) Chemical structures of deuterated (*S*)- and (*R*)-thalidomides, (*S*)-D-Thal and (*R*)-D-Thal, respectively. Atom numbering is shown in the (*S*)-D-Thal chemical structure. The hydrogen atom at the chiral centre C3 atom of the glutarimide moiety is substituted with a deuterium atom. (**b**) Competitive elution assay using thalidomide-immobilized beads coupled with racemic thalidomide. Beads were mixed with extracts from 293 T cells expressing FLAG-HA-CRBN and washed three times with 0.5% NP-40 lysis buffer and bound proteins were eluted with wash buffer containing 1 mM deuterated (*S*)- or (*R*)-thalidomide ((*S*)-D-Thal or (*R*)-D-Thal), (*S*)- or (*R*)-thalidomide ((*S*)-Thal or (*R*)-Thal) or DMSO for the indicated time. The eluate was then analyzed by SDS-PAGE and immunoblotting (IB). (**c**) Inhibitory effects of thalidomide enantiomers on auto-ubiquitylation of FH-CRBN were detected in the presence of MG132. Cells were treated with DMSO or the indicated concentrations of (*S*)-D-Thal or (*S*)-D-Thal for 4 hours prior to harvesting. Full-length blots in (**b**) and (**c**) are presented in Supplementary Fig. 8.

**Figure 2:**
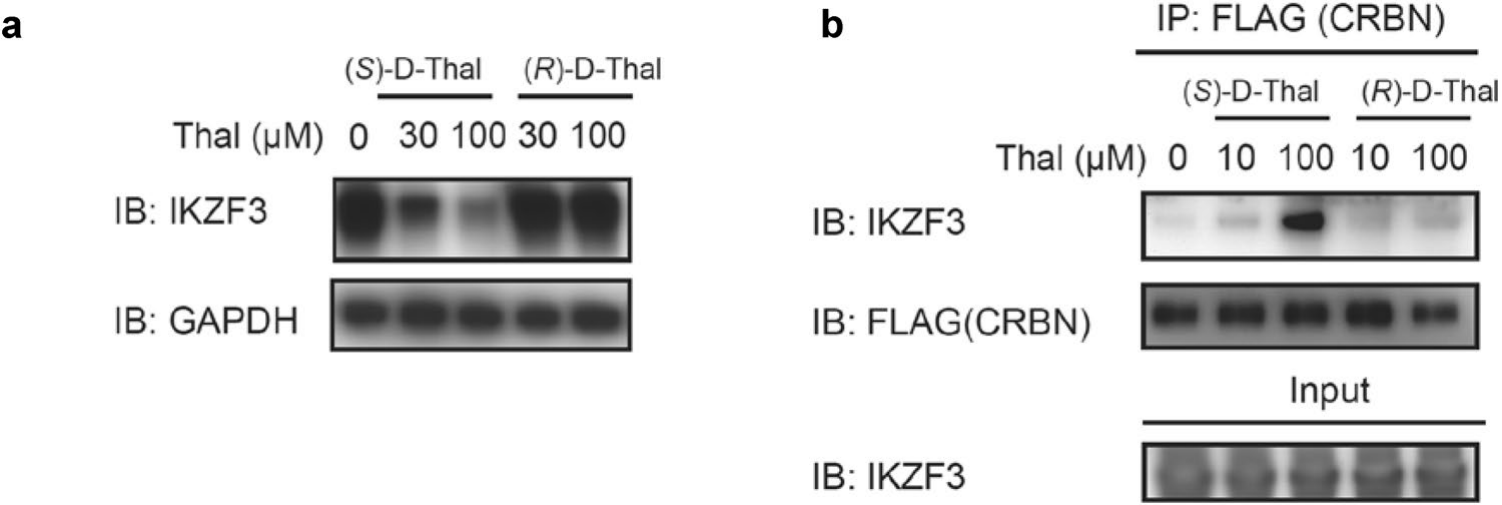
Effects of deuterium-substituted (*S*)- and (*R*)-thalidomides on IKZF3 degradation and CRBN-IKZF3 binding. (**a**) Effects of deuterated (*S*)- and (*R*)-thalidomide enantiomers, (*S*)-D-Thal and (*R*)-D-Thal, on IKZF3 protein levels in MM1S cells after 12-h treatment. IB, immunoblotting. (**b**) Co-immunoprecipitation of FLAG-HA-CRBN in the presence of each deuterated thalidomide enantiomer, (*S*)-D-Thal or (*S*)-D-Thal. IP, immunoprecipitation. Full-length blots in (**a**) and (**b**) are presented in Supplementary Fig. 9.

Since interconversion of thalidomide enantiomers could occur under physiological conditions, special precautions are required to delineate any enantiomer-specific differences in the biological activity of thalidomide. To this end, we synthesized each thalidomide enantiomer with deuterium substitution of the hydrogen atom bonded to the chiral carbon atom (C3, Fig. 1a), hereafter deuterated (*S*)- and (*R*)-thalidomides are referred to as (*S*)-D-Thal and (*R*)-D-Thal in Figs 1 and 2. Deuterated thalidomides have been found to be at least five times more stable than thalidomide with respect to racemization^30^. We initially compared the CRBN-binding affinity of the enantiomers in a competitive binding assay with each deuterated thalidomide enantiomer chemically coupled to ferrite beads (Fig. 1b). CRBN bound to the beads was eluted by the addition of deuterated (*S*)- or (*R*)- thalidomide in a concentration-dependent manner. We found that the binding affinity of CRBN to the (*S*)-enantiomer was ~10-fold stronger than that to the (*R*)-enantiomer. This is consistent with a previous demonstration that non-deuterated (*S*)-thalidomide binds CRBN more tightly than the (*R*)-enantiomer by competition for thalidomide-immobilized beads^28^. The aforementioned conclusion is further supported by results of a cell-based assay, in which deuterated (*S*)-thalidomide inhibited the auto-ubiquitylation activity of the CRBN-containing ubiquitin ligase complex more strongly than deuterated (*R*)-thalidomide (Fig. 1c), and is consistent with previous results utilizing non-deuterated thalidomide^28^. Considering their half-lives of racemization, which occurs in a matter of hours with non-deuterated thalidomide under physiological conditions^11,12,18,19,21,24,25^, only a very small fraction of each enantiomer is thought to be racemized during the assay. To confirm this hypothesis, we analyzed the enantiomeric purities of all the thalidomides used before and after the experiments. The purities were monitored by HPLC (DAICEL Chiralpak IA, 4.6 × 250 mm, MeOH = 100%, flow rate 1.0 ml/min, λ = 254 nm). (Supplementary Table 1 and Supplementary Fig. 1). No significant racemization was detected for the non-deuterated thalidomides and no racemization was detected for the deuterated thalidomides. These results are consistent with a previous report indicating that the racemization of thalidomide is slow under lower or neutral pH conditions, while the racemization is accelerated under higher pH values^30^. The half-life of racemization of (*S*)-thalidomide was 31.8 h at 6.18 pH and 29.9 h at pH 7.78 (at 37 °C), while that of deuterated (*S*)-thalidomide was 156.3 h at pH 6.18 and 59.5 h at pH 7.78^30^. The pH values of the medium solutions used in the current experiments were 5.76 for the Zebrafish E3 medium (5 mM NaCl, 0.17, mM KCl, 0.33 mM MgSO_4_, and 0.33 mM CaCl_2_), 6.54 for the DMEM (Nacalai) Auto-Ub medium and 7.05 for the RPMI1640 MM1S medium, suggesting that both thalidomide and deuterated thalidomide are rather stable toward racemization in each medium used in our assays.

We also determined the dissociation constants *K*_D_ for the binding of non-deuterated (*S*)- and (*R*)-thalidomide to human and mouse CRBN TBDs by isothermal titration calorimetry (ITC). The results show that (*S*)-thalidomide binds TBD 6- or 9-fold more tightly than (*R*)-thalidomide in terms of the *K*_D_ values (Supplementary Fig. 2). These results collectively suggest that differences in the pharmacological activity of the enantiomers are determined, at least in part, by their differential binding affinity to CRBN.

### Deuterated (*S*)*-* and (*R*)- thalidomides showed differences in IKZF degradation

Recent studies have shown that lenalidomide and pomalidomide directly bind CRBN and promote the recruitment of pseudo-substrates Ikaros (IKZF1) and Aiolos (IKZF3) to the E3 complex containing CRBN, thus leading to ubiquitylation and degradation^31,32^. We set out to determine whether enantiomers exert differential effects on the down-stream substrate IKZF1/3. We examined IKZF3 degradation in MM1S cells with deuterated (*S*)- and (*R*)-thalidomides (Fig. 2a). The breakdown of IKZF3 was observed in the presence of deuterated (*S*)-thalidomide ((*S*)-D-Thal in Fig. 2a). However, no discernible breakdown of IKZF3 was observed with deuterated (*R*)-thalidomide. Furthermore, we performed a co-immunoprecipitation assay for CRBN binding to IKZF3 in the presence of deuterated (*S*)- or (*R*)-thalidomide (Fig. 2b). We observed strong CRBN binding to IKZF3 in the presence of deuterated (*S*)-thalidomide. On the other hand, the effect by deuterated (*R*)-thalidomide on the binding was very weak. These results support the notion that the binding affinity of CRBN to the (*S*)-enantiomer was stronger than that to the (*R*)-enantiomer and, that the difference in binding affinity affects degradation of the down-stream substrates.

### Common binding model of (*S*)- and (*R*)-thalidomides to the tri-Trp pocket

**Figure 3:**
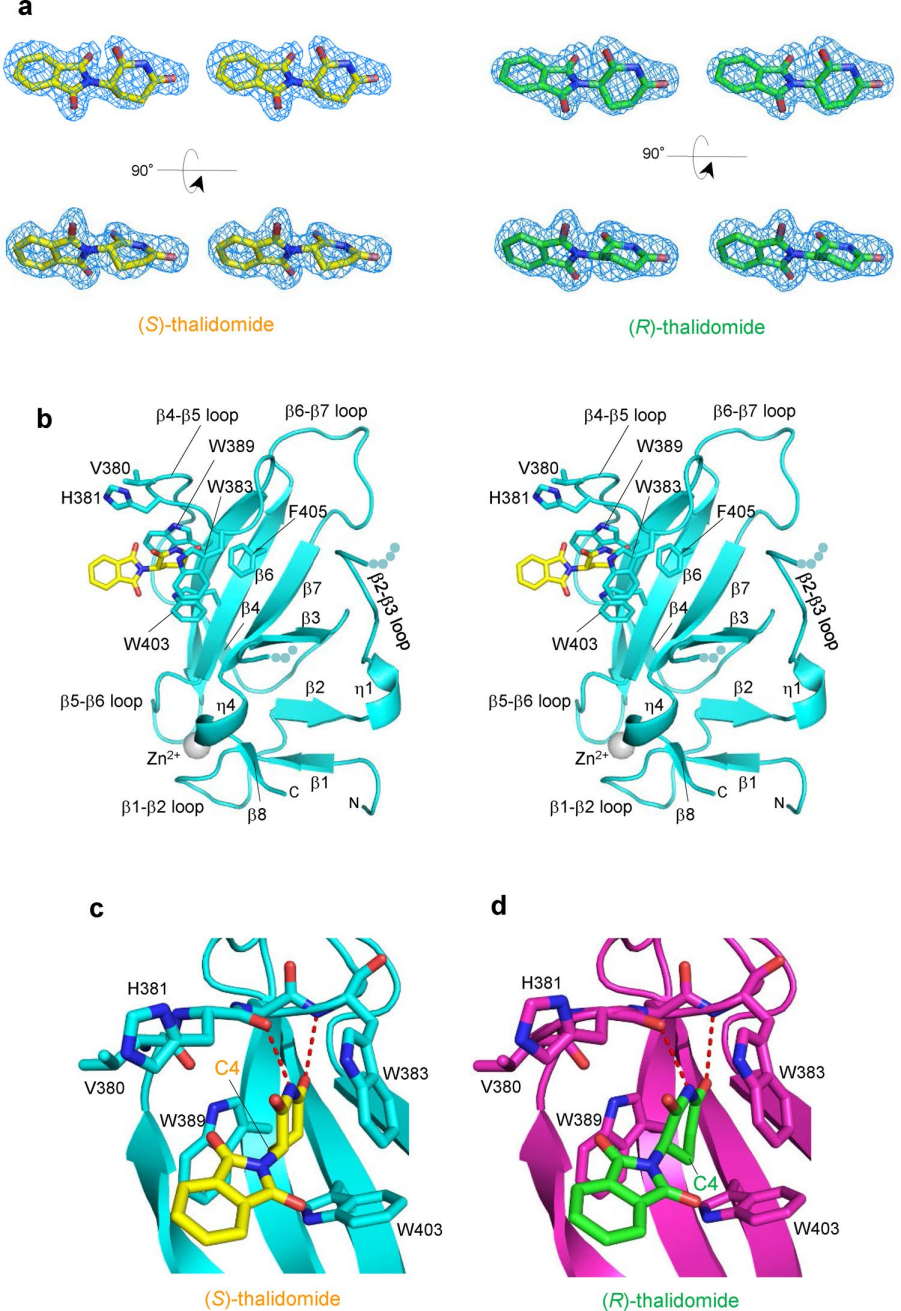
(*S*)- and (*R*)-thalidomides bound to CRBN TBD. (**a**) Stereo views of (*S*)-thalidomide (yellow) and (*R*)-thalidomide (green) molecules bound to mouse CRBN TBD in the crystal with composite omit maps (2mFo-DFc) shown in 1σ contour (blue). (**b**) Stereo view of mouse CRBN TBD (cyan) bound to (*S*)-thalidomide (yellow). Side chains of residues at the tri-Trp pocket are shown as stick models. **(c)** Close-up view of a (*S*)-thalidomide molecule (yellow) bound to the tri-Trp pocket (cyan). Residues forming the tri-Trp pocket are shown as stick models (cyan for C, blue for N, and red for O). The (*S*)-thalidomide molecule is shown as a stick model (yellow for C, blue for N, and red for O). Two hydrogen bonds formed between the protein and the glutarimide moiety of thalidomide are represented as red dotted lines. **(d)** As in **c** but for (*R*)-thalidomide (green for C, blue for N, and red for O) bound to the tri-Trp pocket (magenta).

**Figure 4:**
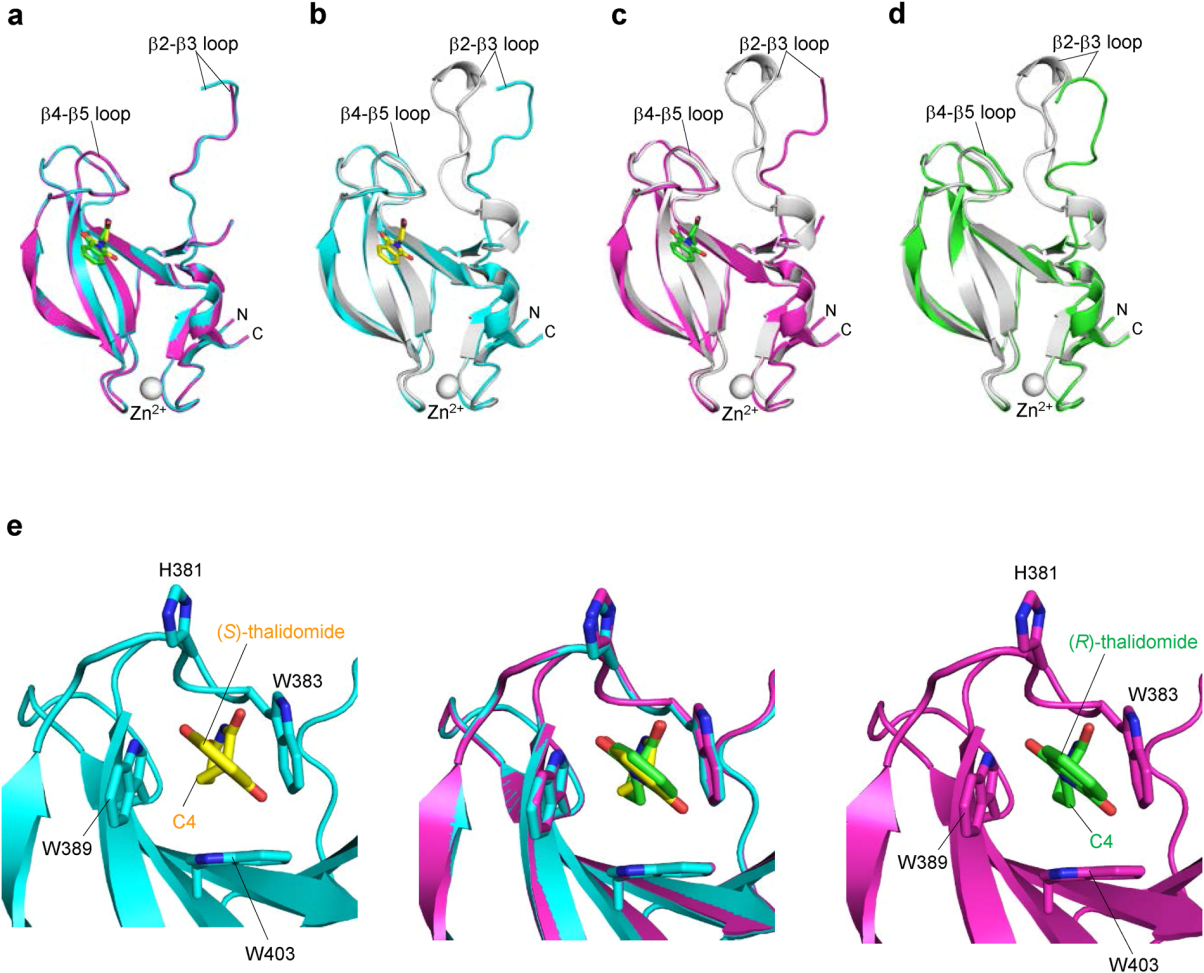
Overall structural comparison of CRBN TBDs in the free and thalidomide-bound forms. (**a**) Comparison of the (*S*)- (cyan) and (*R*)- (magenta) thalidomide-bound forms of CRBN TBD structures. The structural overlay shows no significant structural differences with a small root-mean-square (rms) deviation (0.13 Å). (**b**) Comparison of the (*S*)-thalidomide-bound (cyan) form with the free form^28^ (grey, PDB code 3WX2) of CRBN TBD structures. The rms deviation is 0.45 Å for C_α_ carbon atoms except for the mobile β2-β3 loop (residues 344–359). (**c**) Comparison of the (*R*)-thalidomide-bound (magenta) and free (grey) forms of CRBN TBD structures. The structural overlay shows a large structural deviation in the mobile β2-β3 loop (residues 344–359), although the remainder of the domain cores are similar with a relatively small rms deviation (0.46 Å). (**d**) Two crystallographically independent molecules A (grey) and B (green) of CRBN TBD in the free form^28^ superimposed on each other. Nine residues (351–359) of the flexible β2-β3 loop of molecule B were invisible in the current map. The rms deviation is 1.82 Å for all C_α_ carbon atoms and 0.34 Å for the core domain without the mobile β2-β3 loop (residues 341–361). (**e**) Structural comparison of the tri-Trp pockets accommodating (*S*)-thalidomide (yellow) and (*R*)-thalidomide (green). *Middle*, superposition of (*S*)- and (*R*)-thalidomide-bound tri-Trp pockets. *Left*, A close-up view of the tri-Trp pocket of mouse CRBN TBD (cyan) bound to (*S*)-thalidomide (yellow). *Right*, a close-up view of the tri-Trp pocket of mouse CRBN TBD (magenta) bound to (*R*)-thalidomide (green). Side chains of key residues forming the tri-Trp pocket are shown as stick models.

In an effort to clarify the differences in CRBN binding at the structural level, we determined the structure of mouse CRBN TBD bound to (*S*)- or (*R*)-thalidomide and refined the structures at 1.8 Å and 2.0 Å resolution, respectively (Tables 1 and 2). Both structures gave clear electron densities for the bound thalidomide molecules (Fig. 3a). CRBN TBD consists of eight β-strands (β1-β8) with a single zinc ion, which is coordinated by four conserved cysteine residues from two CXXC motifs located in the β1-β2 and β5-β6 loops (Fig. 3b and Supplementary Fig. 3). The central β-sheet (β4-β5-β6-β7-β3) contains the tri-Trp pocket formed by Trp383 (β4-β5 loop), Trp389 (β5-strand) and Trp403 (β6-strand). (*S*)-thalidomide binds this pocket so that the glutarimide ring is docked into the tri-Trp pocket, and the phthalimido group is located outside of the pocket (Fig. 3b). Inside the pocket, the glutarimide ring, which is sandwiched between Trp383 and Trp389, makes nonpolar contacts with Trp403 forming the floor of the pocket and forms two hydrogen bonds with the protein: the glutarimide 6-carbonyl group forms a hydrogen bond to the main-chain amide of Trp383 and the glutarimide 1-imino group (NH) forms a hydrogen bond to the main-chain carbonyl group of His381 (Fig. 3c). These characteristics of the binding mode are identical to those observed for (*R*)-thalidomide (Fig. 3d) and do not differ from those detailed in previous reports^28,29^. No significant structural differences were found between the overall structures of TBDs bound to (*S*)- and (*R*)-thalidomide, as reflected in the extremely small root-mean-square (rms) deviation (0.13 Å) (Fig. 4a). Additionally, these structures are similar to the free forms^28^ (0.45–0.46 Å) and other reported structures^28,29^ (Fig. 4b,c), although the β2-β3 loop displays conformational flexibility with relatively high temperature factors, as observed in the free forms^28^ (Fig. 4d). In addition to the similarity in the overall structures, the thalidomide-binding sites also exhibit high similarity without any significant structural deviation between the (*S*)- and (*R*)-thalidomide-bound forms (Fig. 4e).

**Table 1:** Crystallographic statistics of the CRBN TBD bound to thalidomides.

	(*S*)-thalidomide	(*R*)-thalidomide	(*RS*)-thalidomide (racemic)
Space group	*R*3 (H)	*R*3 (H)	*R*3 (H)
Unit cell *a* = *b* = (Å)	201.90	202.24	201.38
*c* = (Å)	123.61	123.82	123.36
γ = (°)	120	120	120
Wavelength (Å)	1.28268	0.9000	1.0000
Resolution range^*a*^ (Å)	50–1.8	50–2.0	50–2.0
(Outer shell)	(1.86–1.8)	(2.05–2.0)	(2.07–2.0)
Completeness (%)	100 (100)	99.9 (99.9)	100 (100)
Reflections^b^			
Oscillation range (°)	180	180	180
Measured	974,617	752,877	723,800
Unique	173,436	130,317	125,695
Multiplicity	5.6	5.8	5.8
Mosaicity (°)	0.38	0.41	0.33
*I*/σ(*I*)	17.8 (3.1)	20.6 (3.9)	20.6 (4.6)
*R*_merge_(%)	8.1 (50.0)	7.8 (49.3)	7.8 (41.0)

^a^Values in parentheses are for the highest-resolution shell.

^b^Data were collected at SPring-8 beamline BL44XU with a MX225HE detector (each 1° oscillation with 1 s exposure time for the (*S*)-thalidomide-bound form, 0.5° oscillation with 1.2 s exposure time for the (*R*)-thalidomide-bound form and each 1° oscillation with 1 s exposure time for the (*RS*)-thalidomide-bound form) at 100 K.

**Table 2:** Structural refinement statistics of the CRBN TBD bound to thalidomides.

	(*S*)-thalidomide	(*R*)-thalidomide	(*RS*)-thalidomide (racemic)
*R*_work_/*R*_free_^a^ (%)	18.8/21.6	18.7/21.1	18.9/21.0
Number of atoms	13,621	13,077	13,548
CRBN molecules	16	16	16
residues	1,505	1,500	1,505
Thalidomide	16	16	16
Zinc ions	16	16	16
Sulphate ions	30	30	30
Water molecules	1,313	809	1,240
Averaged B-factors (Å^2^)			
CRBN molecules	27.8	32.4	27.6
Thalidomide	26.9	37.3	28.1
Zinc ions	27.8	27.7	24.8
Sulphate ions	43.9	46.6	42.8
Water molecules	40.9	42.2	40.9
R.m.s.d. from ideal values			
bonds (Å)	0.005	0.005	0.005
angles (°)	1.011	1.029	1.066
Ramachandran plots (%)			
Favored	98.8	98.8	98.7
Allowed	1.2	1.2	1.3
Outliers	0	0	0

^a^*R*_free_ was calculate on a random 5% reflections of the data.

The observed high similarity in the structures of the thalidomide-binding sites, the binding modes, and overall TBD structures of our complexes imply that other factors are responsible for the differences in the binding affinity of (*S*)- and (*R*)-thalidomides.

### Conformational differences between bound (*S*)- and (*R*)-thalidomides

The electron density maps of our complex structures are sufficiently clear to define the conformation of the bound thalidomide molecules (Fig. 3a). We then examined the thalidomide conformation in the CRBN-bound state. Our crystals contain 16 crystallographically independent CRBN TBD-thalidomide complexes in the asymmetric unit (see the Experimental section). We found that the 16 (*S*)-thalidomide molecules bound to CRBN TBD display essentially the same conformation with a small averaged rms deviation of 0.056 Å (Supplementary Fig. 4a). Similarly, the 16 (*R*)-thalidomide molecules display essentially the same conformation, which is distinct from that of (*S*)-thalidomide as described below, and exhibit a small averaged rms deviation of 0.074 Å (Supplementary Fig. 4b). These results suggest that (*S*)- and (*R*)-thalidomide molecules bound to CRBN have a defined conformation but no variable conformations.

**Figure 5:**
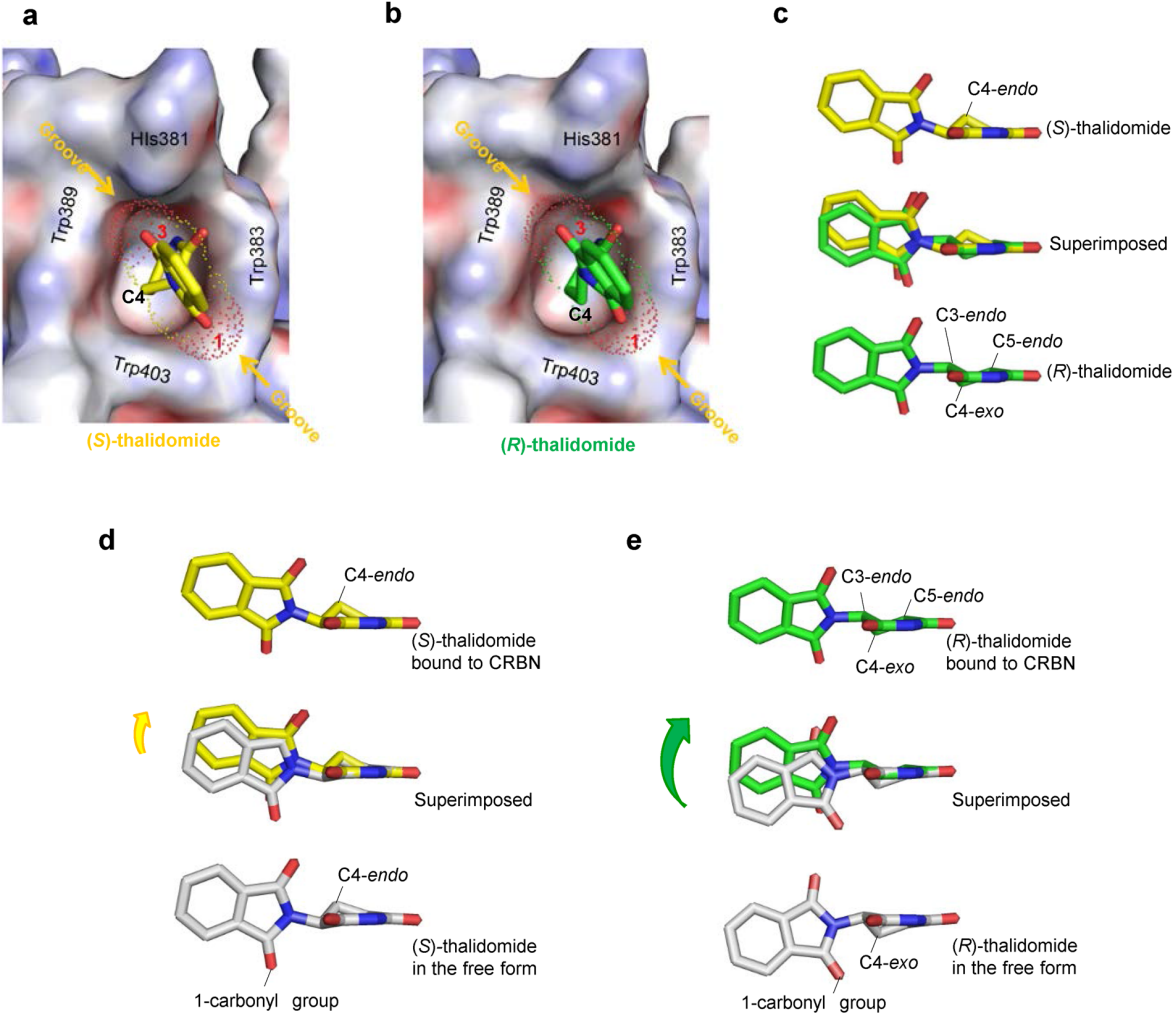
Comparison of the bound thalidomide conformations. (**a**) Phthalimido carbonyl groups of (*S*)-thalidomide docked into grooves formed at the entrance of the tri-Trp pocket. (*S*)-thalidomide (yellow stick model) bound to the Tri-Trp hole of CRBN TBD. The phthalimido 1-carbonyl group (labelled with red 1) is docked into the groove formed by the side chains of Trp383 and Trp403. The phthalimido 3-carbonyl group (labelled with red 3) is docked into the groove formed by Trp389 and His381 side chains. The van der Waals surfaces of the two carbonyl groups are shown with dots and labelled with red atom numbers. The grooves for docking with the phthalimido carbonyl groups are indicated with orange arrows. (**b**) As in **a**, but for (*R*)-thalidomide (green stick model) bound to the tri-Trp pocket of the CRBN TBD. (**c**) Comparison of CRBN-bound thalidomide conformations. (*S*)-thalidomide superimposed onto (*R*)-thalidomide with overlapping imide groups, which are linked to CRBN by direct hydrogen bonds and polar interactions. The puckered C4 atoms are separated from each other by 1.0 Å and the puckered C3 atoms by 0.5 Å. The phthalimido groups are shifted from each other by a maximum of 1.0 Å. (**d**) Comparison of the glutarimide ring conformations of thalidomide molecules in the CRBN-bound and free states in crystals. CRBN-bound (*S*)-thalidomide (yellow) is superimposed onto the free form (grey) of (*S*)-thalidomide in the racemic thalidomide crystal (34) with imide groups overlapped. The glutarimide ring of CRBN-bound (*S*)-thalidomide displays the typical C4-*endo* puckered conformation. This conformation is similar to the glutarimide ring of free (*S*)-thalidomide, which displays a slightly twisted C4-*endo* puckered conformation. The phthalimido group of the CRBN-bound form exhibits a displacement (0.5 Å for the carbonyl groups) from that of the free form with overall rms deviation of the phthalimido groups of 0.9 Å. (**e**) As in (**d**) but showing (*R*)-thalidomides. The glutarimide ring of CRBN-bound (*R*)-thalidomide (green) displays a twisted (C3-*endo-*C4-*exo*-C5-*endo*) conformation. This highly twisted conformation is in sharp contrast with free (*R*)-thalidomide (grey) in the racemic thalidomide crystal (34). In contrast to (*S*)-thalidomide, the phthalimido group of CRBN-bound (*R*)-thalidomide exhibits marked displacement (1.7 Å for the 1-carbonyl group) relative to the free form with large overall rms deviation (1.4 Å) of the phthalimido groups.

In both enantiomeric structures, the phthalimide ring is tilted against the glutarimide ring by 75° so that two protruding carbonyl groups of the phthalimide ring are docked into grooves at the pocket entrance: the phthalimido 1-carbonyl group into the groove between Trp383 and Trp403, and the 3-carbonyl group into the groove between Trp389 and His381 (Fig. 5a,b). Thus, the phthalimide ring is locked at the pocket entrance and the rotational conformation of the phthalimide ring around the C-N bond between the phthalimide and glutarimide rings is the same in both enantiomers. Overlay of the bound (*S*)- and (*R*)-thalidomide molecules shows good overlap of the molecules with a relatively small shift in orientation of the phthalimide ring (Fig. 5c). This shift is caused by the different nature of the glutarimide ring conformation. The glutarimide ring of (*S*)-thalidomide displays a C4-*endo* puckered conformation, a relaxed and stable five-membered ring conformation, in which the C4-carbon atom is displaced from the plane formed by the rest of the ring atoms. This puckered form closely resembles that observed in the isolated free form of thalidomide^33,34^ (Fig. 5d). Compared to the free form, the CRBN-bound form has a small shift (0.5 Å) of the phthalimide ring toward the *endo* direction (middle in Fig. 5d) so as to fit the grooves of the entrance of the tri-Trp pocket (Fig. 5a).

**Figure 6:**
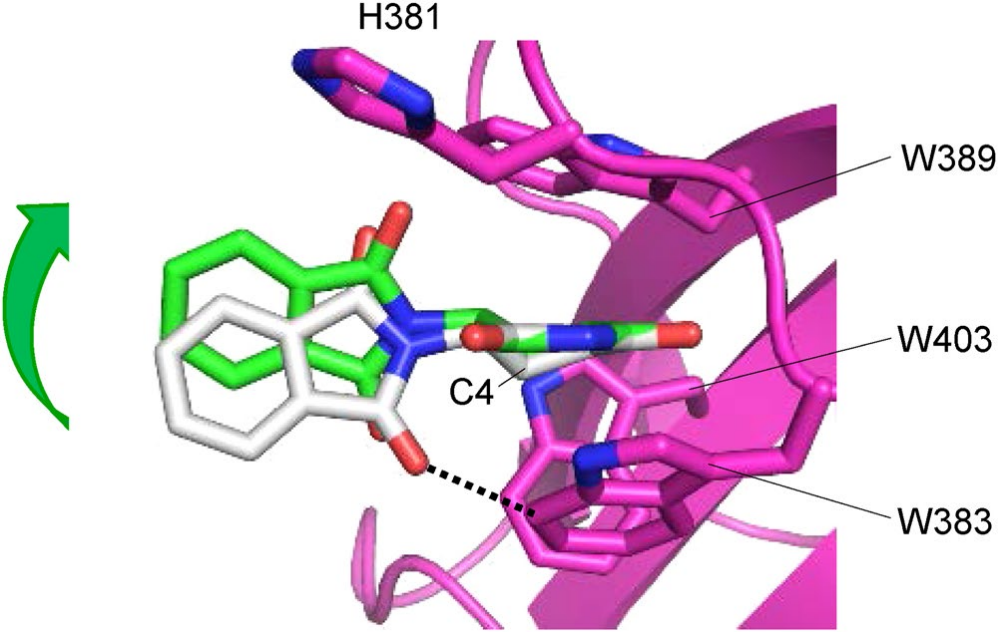
Comparison of (*R*)-thalidomides in the free form and in the CRBN-bound form found in the complex crystal. Superposition of the free form of (*R*)-thalidomide (grey) on the CRBN-bound form of (*R*)-thalidomide (green) found in the crystal. The free form of (*R*)-thalidomide has its glutarimide ring in a C4-*exo* conformation, while (*R*)-thalidomide (green) bound to the tri-Trp pocket of CRBN displays a twisted conformation (see text). The C4-*exo* conformation of the free form causes serious steric clash between the phthalimido-1-carbonyl group and the ring carbon atom of Trp383 (2.6 Å indicated by a dotted line). Additionally, short contacts (~3.6 Å) were found between the C4-carbon atom of the glutarimide moiety and ring carbons of Trp383. The twisted conformation of the bound (*R*)-thalidomide avoids this clash by shifting the phthalimide ring toward the *endo* direction so that the clashes are relaxed (4.0 Å and 3.8 Å, respectively) in the twisted form.

In sharp contrast to the relaxed form of the bound (*S*)-thalidomide, CRBN-bound (*R*)-thalidomide has the glutarimide ring in a twisted conformation, which is distinct from the stereochemically relaxed C4-*exo* puckered conformation of (*R*)-thalidomide (Fig. 5e), suggesting that bound (*R*)-thalidomide is structurally constrained in a metastable conformational state. The twisted conformation is forced primarily by the contacts between the phthalimide ring and the entrance of the tri-Trp pocket and accompanies a large displacement (1.7 Å) of the phthalimide ring toward the *endo* direction (middle in Fig. 5e). This displacement needs to avoid steric clash of the phthalimido-1-carbonyl group against the groove formed by Trp403 and Trp383, and enables the protruding carbonyl groups to dock into the grooves (Fig. 5b). To visualize the steric hindrance, we superimposed the free form of (*R*)-thalidomide onto the CRBN-bound form of (*R*)-thalidomide found in the crystal (Fig. 6). We found that the C4-*exo* conformation of the free form causes serious steric clash (2.6 Å) of the phthalimido-1-carbonyl group against the ring carbon atom of Trp383 (indicated by a dotted line in Fig. 6). The twisted conformation of the bound (*R*)-thalidomide avoids this clash by shifting the phthalimide ring toward the *endo* direction (1.7 Å, green arrow). Moreover, close inspection of the glutarimide ring puckering by modelling revealed that (*R*)-thalidomide in the C4-*exo* puckered glutarimide ring conformation causes steric clash of the C4-carbon atom against Trp383. To avoid this clash, the glutarimide ring is also forced to adopt a twisted conformation. All of these relatively small but significant differences in the binding mode between (*S*)- and (*R*)-thalidomide molecules suggest that the (*S*)-thalidomide bound state of the thalidomide-CRBN complex is more stable than the (*R*)-thalidomide bound state. As with the previous structures^28,29^, our structure of thalidomide-bound CRBN TBD prepared with racemic thalidomide revealed that the bound thalidomide molecules are all (*S*)-enantiomers (Supplementary Fig. 5). This is also consistent with the notion that CRBN preferentially binds the (*S*)-enantiomer.

### Dependence of thalidomide-induced teratogenicity on chirality

**Figure 7:**
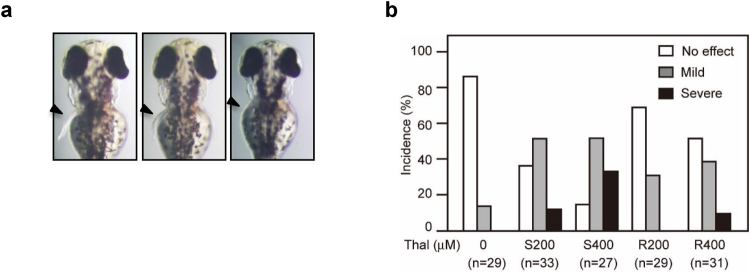
(*S*)-thalidomide treatment causes more sever developmental defects in zebrafish than (R)-thalidomide. (**a**) Dorsal views of pectoral fins of 72-hpf embryos. Fins are indicated by arrowheads. The teratogenic effects resulting from treatment with thalidomide are classified into three categories. Fins that stretched out from the body wall and were more than 85% in length compared to control fins were defined as “no effect.” Fins that stretched out but were shortened to 60–85% of control length were defined as “mild” phenotypes. Fins that showed disc-like morphology and were shortened to less than 60% of control length were defined as “severe” phenotypes. (**b**) Incidence of pectoral fin malformations. Treatments with 200 μM or 400 μM (*S*)-thalidomide (S200 and S400, respectively) induce more severe effects than treatments with 200 μM or 400 μM (*R*)-thalidomide (R200 and R400, respectively) in 72-hpf embryos.

In an effort to examine the effects of thalidomide enantiomers on zebrafish development, we transferred dechorionated embryos to media containing different concentrations of thalidomide at 2 hours post fertilization (hpf) and allowed them to develop for 3 days. It was apparent that with thalidomide-treated embryos, the development of pectoral fins and otic vesicles was disturbed (Fig. 7a). Treatment with 200 μM (*S*)-thalidomide induced severe defects on fins, whereas treatment with 200 μM (*R*)-thalidomide resulted in no discernible severe defects (Fig. 7b). At higher concentrations (400 μM) of drug, (*S*)-thalidomide induced defects in more than 80% of fish with a higher population of defective embryos, whereas (*R*)-thalidomide induced defects in only 50% of fish with a lower population of defects. Thus, (*S*)-thalidomide exerts greater teratogenic effects on fin development of zebrafish, which is consistent with the results of our binding and ubiquitylation-inhibition assays using deuterium-substituted thalidomide enantiomers (Fig. 1), and also the results of the complex structures between TBD and each thalidomide enantiomer.

## Discussion

Based on our complex structures, we are now able to assess the CRBN-binding affinity of thalidomide derivatives. The glutarimide ring in a relaxed six-membered ring conformation is important for CRBN binding with the imide group, serving as both a hydrogen donor and acceptor. Based on the chemical nature of the glutarimide ring, five carbon atoms (C1-3, C5 and C6) of the ring favor an in-plane arrangement because of the *sp*2 configuration of two carbonyl carbon atoms (C2 and C6) with the amido (CONHCO) π electron resonance. Thus, ring puckering usually occurs at the C4 carbon atom. In the isolated state, the C4-*endo* conformation of the (*S*)-thalidomide glutarimide ring minimizes the conformational energy by allowing the N-C bond at the chiral C3 atom to be oriented in a stable equatorial conformation, whereas (*R*)-thalidomide favors the C4-*exo* conformer of the glutarimide ring, allowing the N-C bond to adopt an equatorial conformation. In the C4-*endo* conformer of the (*S*)-thalidomide glutarimide ring, the chiral centre tilts the phthalimido group slightly toward the *endo* direction (bottom in Fig. 5d), which is suitable for binding to the tri-Trp pocket of CRBN TBD. Therefore, small adjustment of the orientation of the phthalimido group is sufficient for CRBN binding (middle in Fig. 5d). In the C4-*exo* conformer of the (*R*)-thalidomide glutarimide ring, however, the chiral centre tilts the phthalimido group slightly toward the *exo* direction (bottom in Fig. 5e), which is in the opposite direction required for binding to the tri-Trp pocket. In this case, small adjustment of the orientation of the phthalimido group is insufficient for CRBN binding, and therefore the glutarimide ring pucker of (*R*)-thalidomide needs to be changed to the twist conformation, C4-*exo*-C5-*endo*, to shift the phthalimido group up toward the *endo* direction (middle in Fig. 5e) for CRBN binding with loss of conformational energy. It should be noted that this enantiomer-specific discrimination by the tri-Trp pocket is mediated by hydrogen bonding interactions between the amide group of the glutarimide ring and the inside of the pocket, in addition to contacts between carbonyl groups of the phthalimido group and the entrance of the pocket.

Lenalidomide and pomalidomide have attracted much attention from pharmaceutical scientists and physicians as hopeful IMiDs in the treatment of multiple myeloma and other cancers^11,14^. The homeobox transcription factor MEIS2, has been implicated in various aspects of human development, has been suggested to be an endogenous substrate of CRL4^CRBN^, and IMiDs block MEIS2 from binding to CRL4^CRBN^ ^28^. Contrary to this inhibition of substrate binding, these drugs directly bind CRBN and promote CRL4^CRBN^ binding to IKZF1 and IKZF3, leading to ubiquitylation and degradation^31,32^. Furthermore, in the treatment of myelodysplastic syndrome (MDS) with deletion of chromosome 5q (del(5q)), lenalidomide induces ubiquitylation of casein kinase 1A1 (CK1α) and its degradation^35^. One attractive hypothesis concerning IMiDs drawn from these IMiD-dependent degradations is that IMiDs act as modulators of CRL4^CRBN^ substrate recognition by playing a role as a molecular glue^36^ or an interfacial drug^37^ that specifically links CRBN and substrate proteins through direct interactions with both proteins. The binding mode of IMiDs to CRBN suggests that the phthalimido group should make contact with the substrate proteins. Since the orientation of the phthalimido group of CRBN-bound IMiDs differs somewhat between (*S*) and (*R*)-enantiomers, we speculate that CRBN bound to the (*S*) and (*R*) enantiomers may exhibit different binding affinity to the substrates. The recent structure of the complex between lenalidomide-bound CRBN and CK1α has shown that CK1α binding repositions the phthalimido ring of lenalidomide 2.5 Å toward CRBN residue Glu377^38^. Interestingly, this shift accompanies a movement of the phthalimido group toward the *endo* direction as seen in (*S*)-thalidomide (Fig. 5d), suggesting that the (*S*)-enantiomer is favored for CK1α binding. However, details of the differences between (*S*)- and (*R*)-enantiomers in CK1α binding should be clarified by further studies.

**Figure 8:**
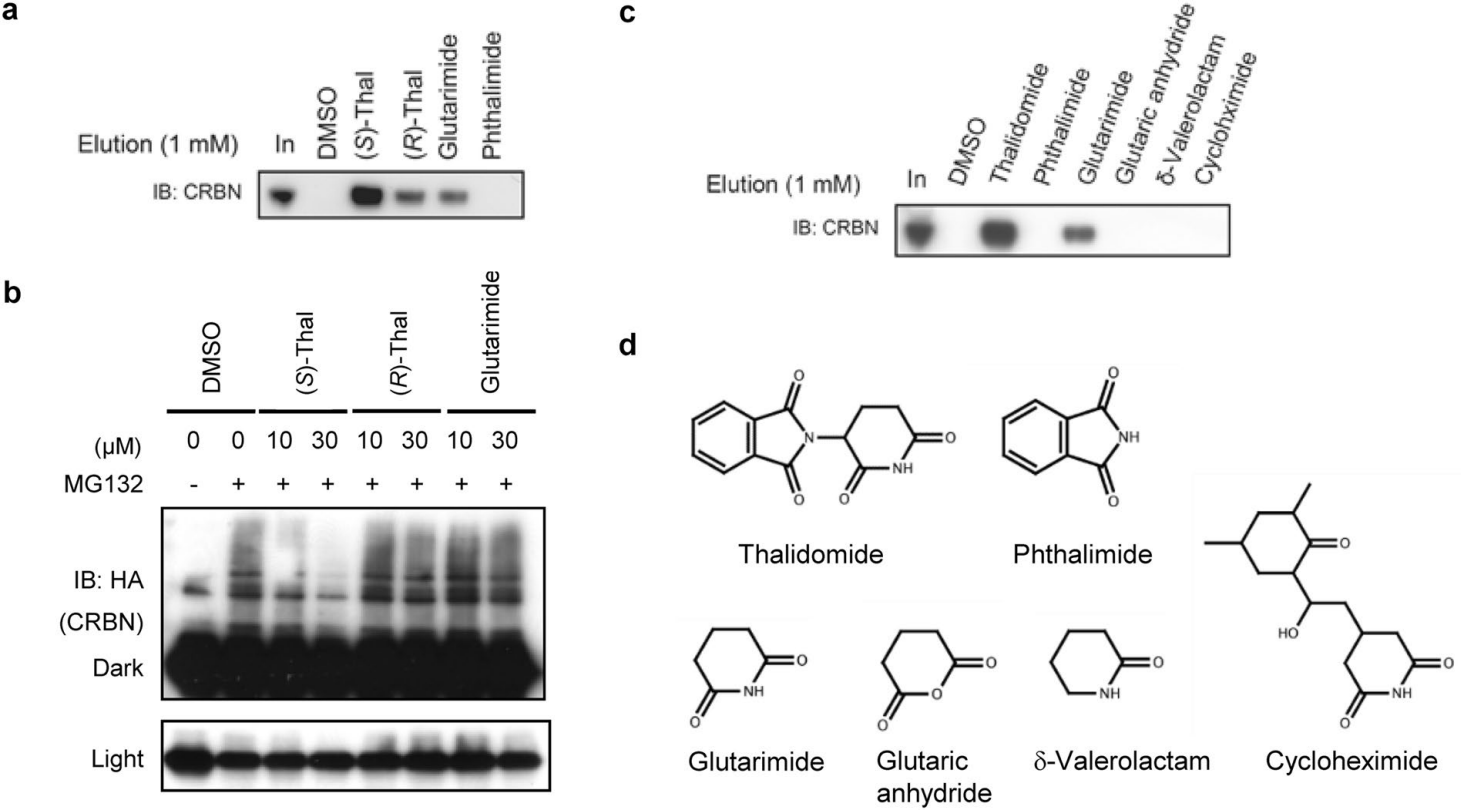
CRBN binding and E3 inhibition by thalidomide derivatives. (**a**) Competitive elution assay with racemic thalidomide-immobilized beads. Glutarimide binds FLAG-CRBN with an affinity similar to that of (*R*)-thalidomide, but weaker than that of (*S*)-thalidomide. Phthalimide exhibits no binding. FLAG-CRBN was processed as in Fig. 1b. (**b**) Effect of glutarimide binding on CRBN auto-ubiquitylation by CRBN-containing E3 ubiquitin ligase CRL4. The method used was that described in Fig. 1c. (**c**) Competition binding assay using thalidomide-immobilized beads. The eluate with 1 mM thalidomide and glutarimide yielded bands, but not with other compounds. The intact glutarimide ring in a relaxed six-membered ring conformation is important for CRBN binding with the imide group serving as both a hydrogen donor and acceptor. Correspondingly, glutarimide still binds CRBN, although phthalimide and glutaric anhydride, which lack the ring amide of glutarimide, exhibited no binding. Both carbonyl groups of glutarimide are important, and the absence of one carbonyl group results in loss of binding (δ-valerolactam). Bulky modification of the glutarimide C4-carbon atom also results in loss of CRBN binding due to steric clash (cycloheximide). (**d**) Chemical structures of compounds utilized in the binding assay. Full-length blots in (**a**), (**b**) and (**c**) are presented in Supplementary Fig. 10.

As with previously reported structures^28,29^, binding to CRBN is primarily mediated by the glutarimide ring. Correspondingly, glutarimide alone is able to bind CRBN and inhibit ubiquitylation to a similar extent as (*R*)-thalidomide (Fig. 8a,b and Supplementary Fig. 6), whereas phthalimide and glutaric anhydride, which lack the ring amide of glutarimide, exhibit no binding (Fig. 8c,d). Both carbonyl groups of glutarimide are important, and the absence of one carbonyl group results in loss of binding (δ-valerolactam in Fig. 8d). Bulky modification of the glutarimide C4-carbon atom also results in loss of CRBN binding due to steric clashes (cycloheximide in Fig. 8d). Upon ingestion, thalidomide undergoes enzymatic and nonenzymatic modification and breakdown, yielding a number of metabolites with a peak plasma concentration at 3–6 h^2,39,40^. The primary metabolic derivatives generated by cytochrome P450 isozymes are derived from 4- or 5-hydroxylation of the phthalimido moiety or 5-hydroxylation of the glutarimide moiety, which subsequently undergo spontaneous hydrolysis^12,41^ (Supplementary Fig. 7). Based on our structural data, metabolites with phthalimido modifications may still bind CRBN, whereas 4- or 5-hydroxylation of the glutarimide moiety is likely to result in loss of binding due to steric clashes. Hydrolysis yields three primary products (I-III in Supplementary Fig. 7) by ring opening followed by further degradation into eight minor products^42–47^. We suggest classifying the hydrolysis processes into three pathways A, B and C. Compounds in pathway A retain the intact glutarimide ring and likely still bind CRBN, while compounds in pathways B and C with opened glutarimide rings do not bind CRBN.

In conclusion, our study provides a structural framework for further investigations on the mechanisms of the pharmaceutical and teratogenic actions of this drug and for the development of more effective IMiDs.

## Methods

### Protein expression and purification

Mouse CRBN TBD (residues 322–430) was cloned into pGEX6P-3 (GE Healthcare) and transformed into *Escherichia coli* strain BL21(DE3) Star (Invitrogen). The *E. coli* cells were cultured at 25 °C in TB-5052 medium^48^ containing 50 μM zinc acetate. The supernatant of the cell lysate was applied to a Glutathione Sepharose 4B resin (GE Healthcare) and resin-bound GST-fusion protein was cleaved from the resin using 4 units/ml HRV3C protease (Merck). Eluted protein fractions were further purified by cation exchange (HiTrap SP HP, GE Healthcare) and gel-filtration (Superdex 75 pg, GE Healthcare) chromatography. Purified CRBN protein sample was dialyzed against a buffer solution containing 0.1 mM thalidomide (SIGMA), 5 mM sodium acetate (pH 6.0) and 10 mM 2-mercaptoethanol (changed three times). Tris-(2-carboxyethyl)-phosphine (TCEP) was added to the protein sample solution up to 10 mM. Purified proteins were analyzed by sodium dodecyl sulphate-polyacrylamide gel electrophoresis (SDS-PAGE) and matrix-assisted laser desorption/ionization time-of-flight mass spectrometry (MALDI-TOF MS; Bruker Daltonics). MALDI-TOF MS of mouse and human TBD domains gave peaks at 12321.2 Da (calculated 12321.1 Da) and 12,456.4 Da (12,477.1 Da), respectively.

### Crystallization

Crystallization conditions were searched for using the sitting-drop vapour diffusion method and Hydra II-Plus-One crystallization robot (Matrix Technology) with a commercial crystallization solution kit. The best crystals of the (*S*)-, (*R*)-, and racemic thalidomide-bound form of mouse TBD were obtained from a 1:1 mixture solution comprising 30 mg/ml of the protein solution (in 5 mM sodium acetate (pH 6.0), 10 mM 2-mercaptoethanol, 10 mM TCEP and 0.1 mM thalidomide) and reservoir solution (100 mM sodium acetate (pH 5.0) and 400–600 mM ammonium sulphate). Crystals appeared within 10 days at 4 °C. The 50–100 μm chunky crystals were transferred into reservoir solution containing 30% glycerol and then flash-cooled in liquid nitrogen.

### Data collection

Diffraction tests of the crystals were performed using a Rigaku R-AXIS VII detector equipped with a Rigaku FR-E X-ray generator. For structure determination, diffraction data of native and SeMet-labelled crystals were collected at 100 K with the MX225HE detector on BL41XU and BL44XU beamlines at the SPring-8 synchrotron facility (Table 1). Diffraction data were processed using the HKL2000 program^49^. Crystals of the three thalidomide-bound forms belong to space group *R*3(H), with *V*_M_ values in the range 2.54–266 Å^3^/Da, suggesting a solvent content of 51–53% assuming 16 proteins in the asymmetric unit.

### Structure determination and refinement

The crystal structures of the thalidomide-bound forms were determined by molecular replacement using the model structure of mouse CRBN TBD (PDB code: 3WX2)^28^ as a search model. The built model was refined through alternating cycles using the Coot^50^ and CNS programs^51^ or REFMAC^52^. For model building and refinements, composite omit maps were calculated with CNS programs^51^ and ligand parameters were generated using PRODRG2^53^. The secondary structures of models were calculated using DSSP^54^. The refinement statistics are summarized in Table 2. These crystals contain 16 molecules in the asymmetric unit. Of these, 15 molecules form 5 trimers similar to those of the free form but with non-crystallographic three-fold axes, while one molecule forms a trimer with symmetry-related molecules related by a crystallographic three-fold axis. No significant structural deviations were found among these molecules with small averaged rms deviations (0.38–0.40 Å). No outliers were flagged in the Ramachandran plots using MolProbity^55^.

### Structural comparison

Proteins and ligands were superposed using the program LSQKAB^56^. Coordinate files of the free form of mouse CRBN TBD^28^ (PDB code: 3WX2) and the free form of thalidomide^33^ (CCDC identifier: 1270378) were obtained from the Protein Data Bank (PDB) and the Cambridge Structural Database (CSD), respectively.

### Antibodies

Anti-mouse monoclonal CRBN antibody was generated in house (epitope 1–18). Aside from this, primary antibodies against FLAG (M2. Sigma), HA (3F10, Roche), IKZF3 (ab139408, Abcam), and GAPDH (3H12, MBL) were used.

### Thalidomide and related compounds

Racemic thalidomide was purchased from Tocris Biosciences. (*S*)- and (*R*)-thalidomides and their deuterated (D-thalidomide) enantiomers were synthesized as previously described^30,57^. Enantiomeric purities were monitored by HPLC (DAICEL Chiralpak IA, 4.6 × 250 mm, MeOH = 100%, flow rate 1.0 ml/min, λ = 254 nm) and are summarized in Supplementary Table 1. Thalidomide and D-thalidomide enantiomers were dissolved in dimethylsulphoxide (DMSO) at room temperature to generate a 100 mM stock solution. Similarly, glutarimide, phthalimide, glutaric anhydride, δ-valerolactam and cycloheximide (Sigma Aldrich) were each dissolved in DMSO.

### *In vivo* binding assay using thalidomide-immobilized beads

Binding assays were performed essentially as previously described^25^. Human CRBN proteins were overexpressed in 293 T cells by transfecting FLAG and haemagglutinin (HA) epitope–tagged (FH)- human CRBN constructs using Lipofectamine 2000 (Invitrogen). 293 T cells were maintained in DMEM (Nacalai Tesque) supplemented with 10% FBS. Cell extracts were incubated with thalidomide-immobilized beads, and bound material was eluted with buffer containing thalidomide, thalidomide-related compounds, or SDS. Lysates (input) and bead-affinity-purified (AP) materials were immunoblotted (IB). Recombinant FLAG-CRBN proteins were expressed in *Sf*9 cells using the Bac-to-Bac system (Invitrogen) and immunopurified using anti-FLAG antibody. Subsequent binding assays were performed as described above.

### *In vitro* ubiquitylation assay

Auto-ubiquitylation of FH-CRBN was detected in the presence of the protease inhibitor MG132 as previously described^25^. Cells stably expressing FH-CRBN were treated for 3 hours prior to harvesting with the proteasome inhibitor MG132 (10 μM) or left untreated. Lysates were incubated with M2 anti-FLAG agarose beads. FH-CRBN was eluted with SDS and then subjected to SDS-PAGE and immunoblotting using anti-HA antibody (3F10, Roche). When indicated, (*S*)- or (*R*)-thalidomide was added to cells 1 hour prior to MG132 addition.

### Thalidomide treatment of zebrafish

Fish were kept at 28.5 °C on a 14-h light/10-h dark cycle, and embryos were obtained by natural matings of adult fish as previously described^25^. Zebrafish embryos were dechorionated prior to thalidomide treatment as follows: At 2 hpf, embryos were incubated in E3 medium containing 2 mg/ml Protease type XIV (Sigma) for 3 min at room temperature and then washed five times with E3 medium. Following dechorionation, embryos were immediately transferred to E3 medium containing thalidomide and further incubated for 24 to 73 h at 28.5 °C. The E3 medium was replaced with medium containing freshly prepared thalidomide every 12 h. Zebrafish (*Danio rerio*) were maintained in accordance with the Animal Research Guidelines at Tokyo Institute of Technology and Tokyo Medical University. The experimental protocol was reviewed and approved by the Animal Research Committee and the method was carried out in accordance with the committee’s approved guidelines.

### IKZF3 degradation assay

MM1S cells were treated with DMSO or deuterated-thalidomide enantiomers for 12 h.

Whole-cell extracts were prepared using RIPA buffer (50 mM Tris-HCl (pH 7.4), 150 mM NaCl, 0.5% DOC, 0.1% SDS and 1% NP-40) and subjected to immunoblot analysis. MM1S cells were maintained in RPMI1640 (Invitrogen) supplemented with 10% FBS.

### Co-immunoprecipitation assay

293 T cells stably expressing FLAG-HA-CRBN were transfected with HA-Aiolos expression vectors. After 48 hours, cells were collected and lysed using 0.5% NP-40 lysis buffer containing 50 mM Tris-HCl (pH 7.4), 150 mM NaCl and 0.5% NP-40. Extracts were incubated with M2 FLAG magnetic beads (Sigma) in the presence of (*S*)-D-thalidomide or (*R*)-D-thalidomide and incubated for 2 h. Following extensive washing three times with 0.5% NP-40 lysis buffer, bound proteins were eluted with free 3x FLAG-peptides (Sigma) and then subjected to immunoblot analysis.

### Binding assay using calorimetry

Binding studies utilizing isothermal titration calorimetry (ITC) were conducted using a calorimeter (MicroCal iTC_200_, GE Healthcare) at 20 °C. Purified proteins were dialyzed overnight in buffer containing 10 mM sodium phosphate (pH 6.8), 150 mM NaCl and 0.5 mM tris(2-carboxyethyl)phosphine (TCEP). Given the poor solubility of the drugs, reverse titration was employed whereby CRBN TBD solution (1.2 mM) was injected (1.5 μl each, 5 min pause) into each drug solution (150 μM). Details of each titration are given in the figure legends of Supplementary Figs 2 and 5. Data fitting was performed using ORIGIN^TM^ software supplied with the instrument.

### Accession code

Protein Data Bank: The atomic coordinates and structure factors for the reported crystal structures are deposited under accession codes 5YJ0 (the (*S*)-thalidomide-bound form of CRBN TBD), 5YJ1 (the (*R*)-thalidomide-bound form) and 5YIZ (the (*S*)-thalidomide-bound form prepared with racemic thalidomide).

## Electronic supplementary material

Supplementary figures
