# Supplementary material for: Structural basis of thalidomide enantiomer binding to cereblon
Source: Sci Rep. 2018 Jan 22;8:1294. doi: 10.1038/s41598-018-19202-7 (PMC5778007; doi:10.1038/s41598-018-19202-7)
Supplement: Supplementary file 1 — Supplementary figures [file 41598_2018_19202_MOESM1_ESM.pdf]

Structural basis of thalidomide enantiomer binding to cereblon

Tomoyuki Mori[1], Takumi Ito[2,3], Shujie Liu[4], Hideki Ando[2], Satoshi Sakamoto[4], Yuki Yamaguchi[4], Etsuko Tokunaga[5], Norio Shibata[5], Hiroshi Handa[2] and Toshio Hakoshima[1]

[1]Structural Biology Laboratory, Nara Institute of Science and Technology, 8916-5 Takayama, Ikoma, Nara 630-0192, Japan
[2]Department of Nanoparticle Translational Research, Tokyo Medical University, Tokyo, Japan.
[3]PRESTO, JST, 4-1-8, Honcho, Kawaguchi, Saitama, 332-0012, Japan
[4]School of Life Science and Technology, Tokyo Institute of Technology, Yokohama 226-8501, Japan.
[5] Department of Nanopharmaceutical Sciences, Nagoya Institute of Technology, Gokiso, Showa-ku, Nagoya 466-8555, Japan

Supplementary Table 1. Monitoring of the enantiomeric purities of thalidomide

	Before drug incubation		After drug incubation	
	S (%)	*R* (%)	*S* (%)	*R* (%)
S-thalidomide (Zebrafish, 12 h)	100	0	96.163	3.837
R-thalidomide (Zebrafish, 12 h)	0	100	0	100
S-D-thalidomide (Auto-Ub, 4h)	100	0	100	0
R-D-thalidomide (Auto-Ub, 4h)	0	100	0	100

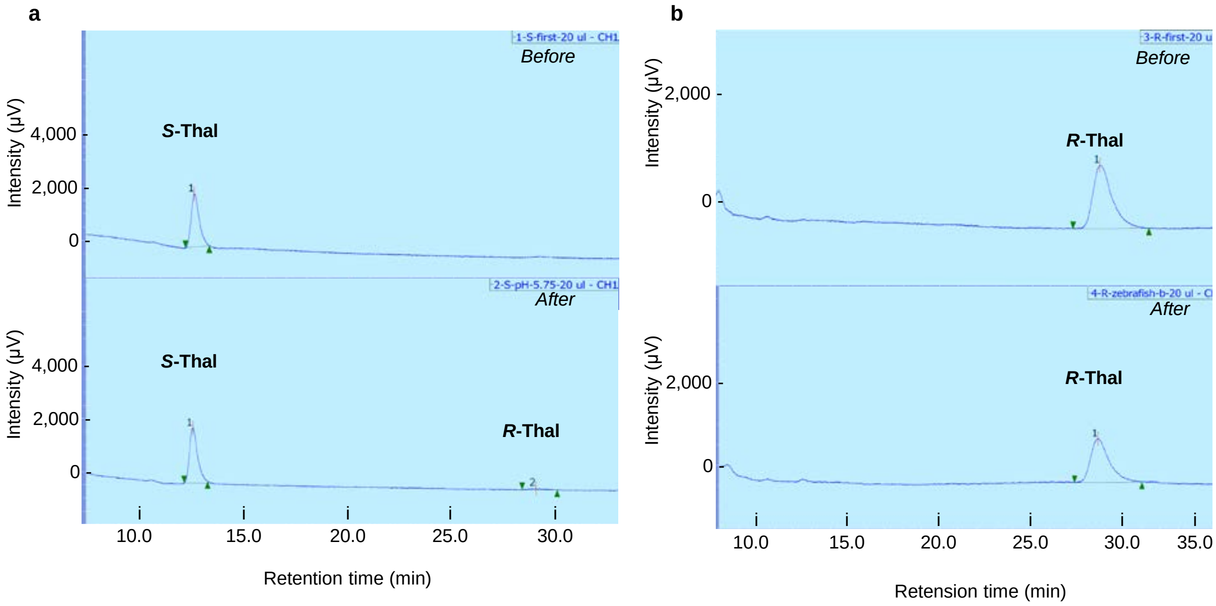

Supplementary Figure 1 (continued)
The enantiomeric purities monitored by HPLC.

c

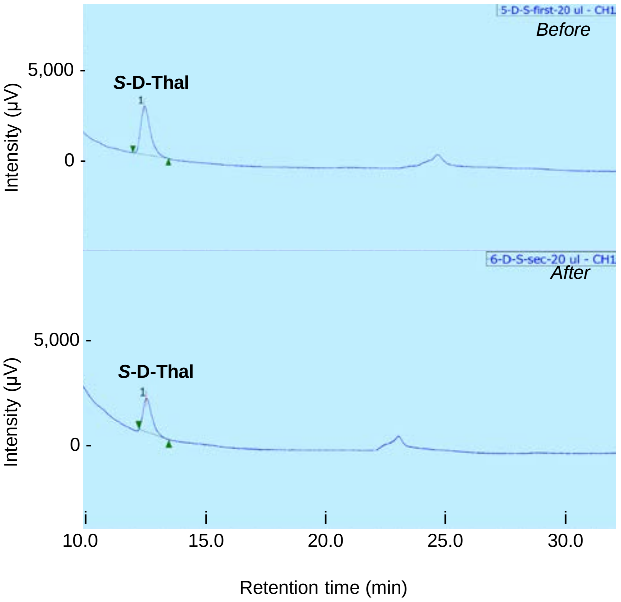

d

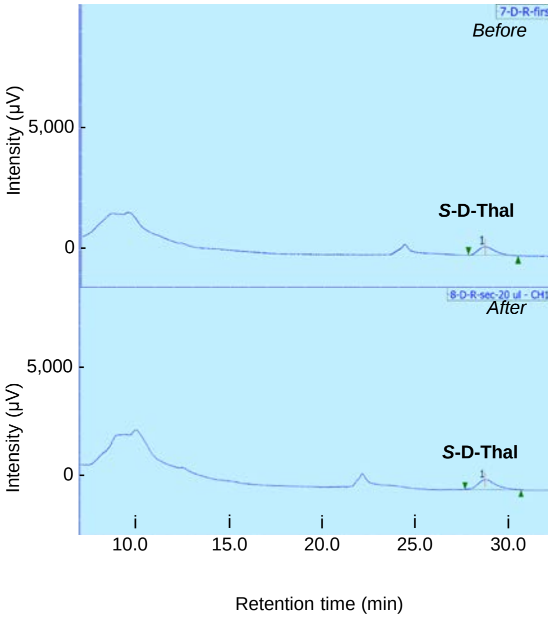

e

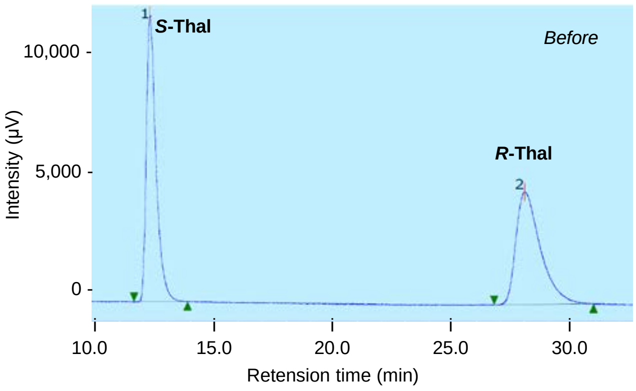

Supplementary Figure 1
The enantiomeric purities monitored by HPLC.

The enantiomeric purities were monitored by HPLC (DAICEL chiralpak IA, 4.6 × 250 mm, MeOH=100%, flow rate 1.0 ml/min, λ=254 nm). Each peak area was integrated between arrow heads (green) shown in the panel.

(**a**) HPLC elution profiles of (*S*)-thalidomide (*S*-Thal) before (*top*) and after (*Bottom*) the experiments of thalidomide treatment of zebrafish. The pH of the medium (5 mM NaCl, 0.17, mM KCl, 0.33 mM $MgSO_4$, and 0.33 mM $CaCl_2$) is 5.76.

(**b**) As in **a**, but for (*R*)-thalidomide (*R*-thal) before (*top*) and after (*Bottom*) the zebrafish experiments (see Supplementary Table 1).

(**c**) As in **b**, but for for deuterated (*S*)-Thalidomide, (*S*)-D-Thal, before (*top*) and after (*Bottom*) the auto-ubiquitylation experiments (see Supplementary Table 1). The pH of the medium of 6.54.

(**d**) As in **c**, but for deuterated (*R*)-Thalidomide, (*R*)-D-Thal. Before the experiment, the peak area is 45,608 µV/sec for *R*-D-Thal but no peak was detected for *S*-D-Thal. After the experiment, the peak area is 31,517 µV/sec for *R*-D-Thal but no peak was detected for *S*-D-Thal.

(**e**) HPLC elution profiles of racemic thalidomide for confirmation of the 1:1 enantiomer ratio. The peak areas are 337,651 µV/sec for *S*-Thal and 337,894 µV/sec for *R*-Thal, suggesting that our racemic thalidomide is a 1.0000:1.0007 racemate.

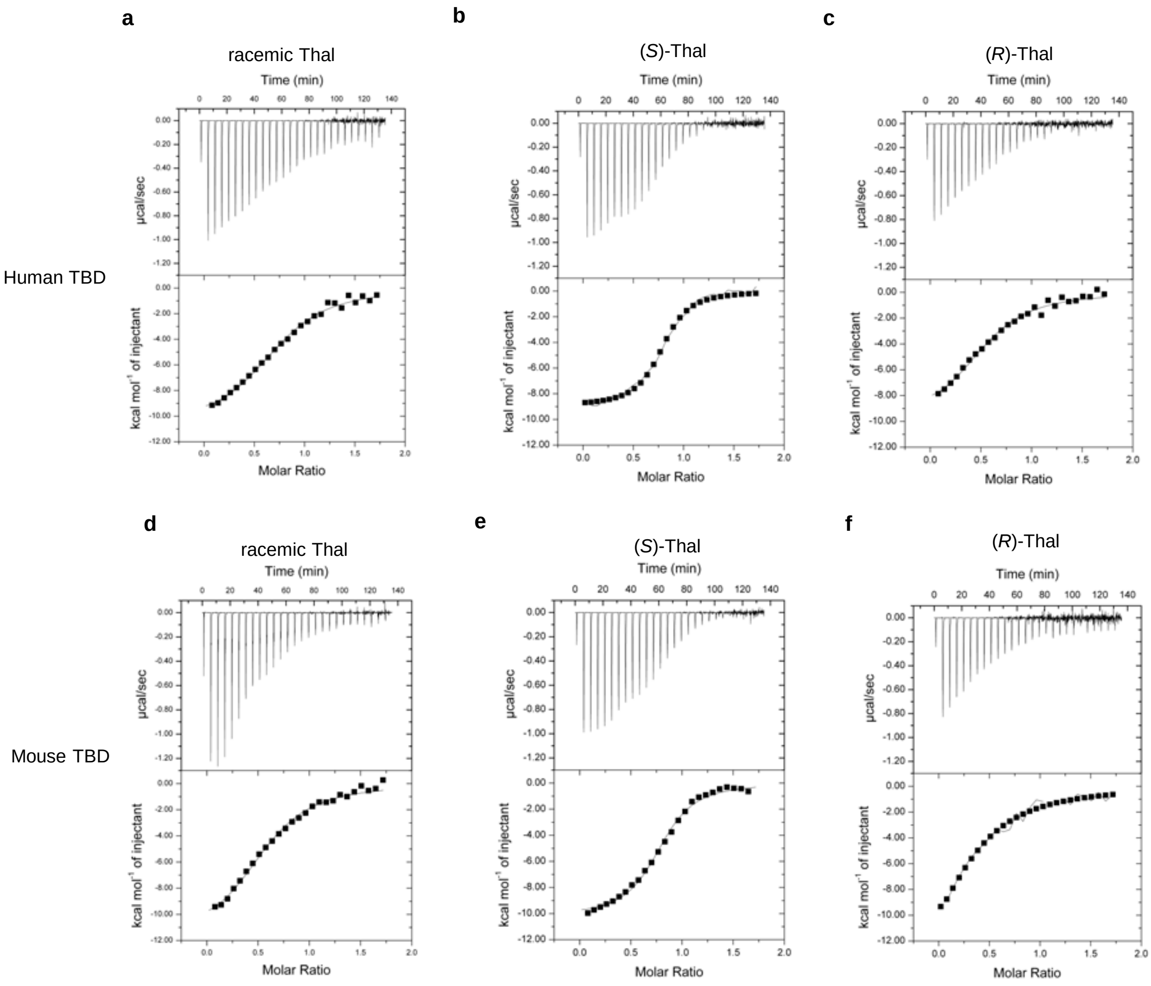

g

	Racemic thalidomide	(*S*)-thalidomide	(*R*)-thalidomide
Human TBD	18.1 ± 1.8 µM	3.5 ± 0.4 µM	20.0 ± 2.9 µM
Mouse TBD	19.5 ± 2.2 µM	5.7 ± 0.5 µM	50.3 ± 11.1 µM

Supplementary Figure 2
Binding assay of thalidomide with TBD of human and mouse CRBN with isothermal titration calorimetry (ITC).

(**a**) Binding of racemic thalidomide to TBD of human CRBN. Reverse titration was performed with injections of 1.5 µl of 1,500 µM TBD into 150 µM racemic thalidomide (250 µl) at 20°C. The injections were performed over a period of 4 sec with a 300-sec interval between injections, and the final concentration reached was 200 µM (26 injections total). TBD exhibited a K_D value of 18.1±1.8 µM, with ΔH and $T\Delta S$ values of -10.7 ± 0.3 and -4.3 kcal/mol, respectively.
(**b**) As in **a**, but for binding of (*S*)-thalidomide (*S*-thal) to TBD of human CRBN. TBD exhibited a K_D value of 3.5 ± 0.4 µM, with ΔH and $T\Delta S$ values of -9.0 ± 0.2 and -1.6 kcal/mol, respectively.
(**c**) As in **a**, but for binding of (*R*)-thalidomide (*R*-thal) to TBD of human CRBN. TBD exhibited a K_D value of 20.0 ± 2.9 µM, with ΔH and $T\Delta S$ values of -9.9 ± 0.5 and -3.6 kcal/mol, respectively.
(**d**) As in **a**, but for binding of racemic thalidomide to TBD of mouse CRBN. TBD exhibited a K_D value of 19.5 ± 2.2 µM, with ΔH and $T\Delta S$ values of -12.0 ± 0.5 and -5.5 kcal/mol, respectively.
(**e**) As in **a**, but for binding of (*S*)-thalidomide (*S*-thal) to TBD of mouse CRBN. TBD exhibited a K_D value of 5.7 ± 0.5 µM, with ΔH and $T\Delta S$ values of -10.2 ± 0.1 and -3.0 kcal/mol, respectively.
(**f**) As in **a**, but for binding of (*R*)-thalidomide (*R*-thal) to TBD of mouse CRBN. TBD exhibited a K_D value of 50.3 ± 11.1 µM, with ΔH and $T\Delta S$ values of -18.5 ± 4.1 and +12.7 kcal/mol, respectively.
(**g**) Summary of the obtained K_D values.

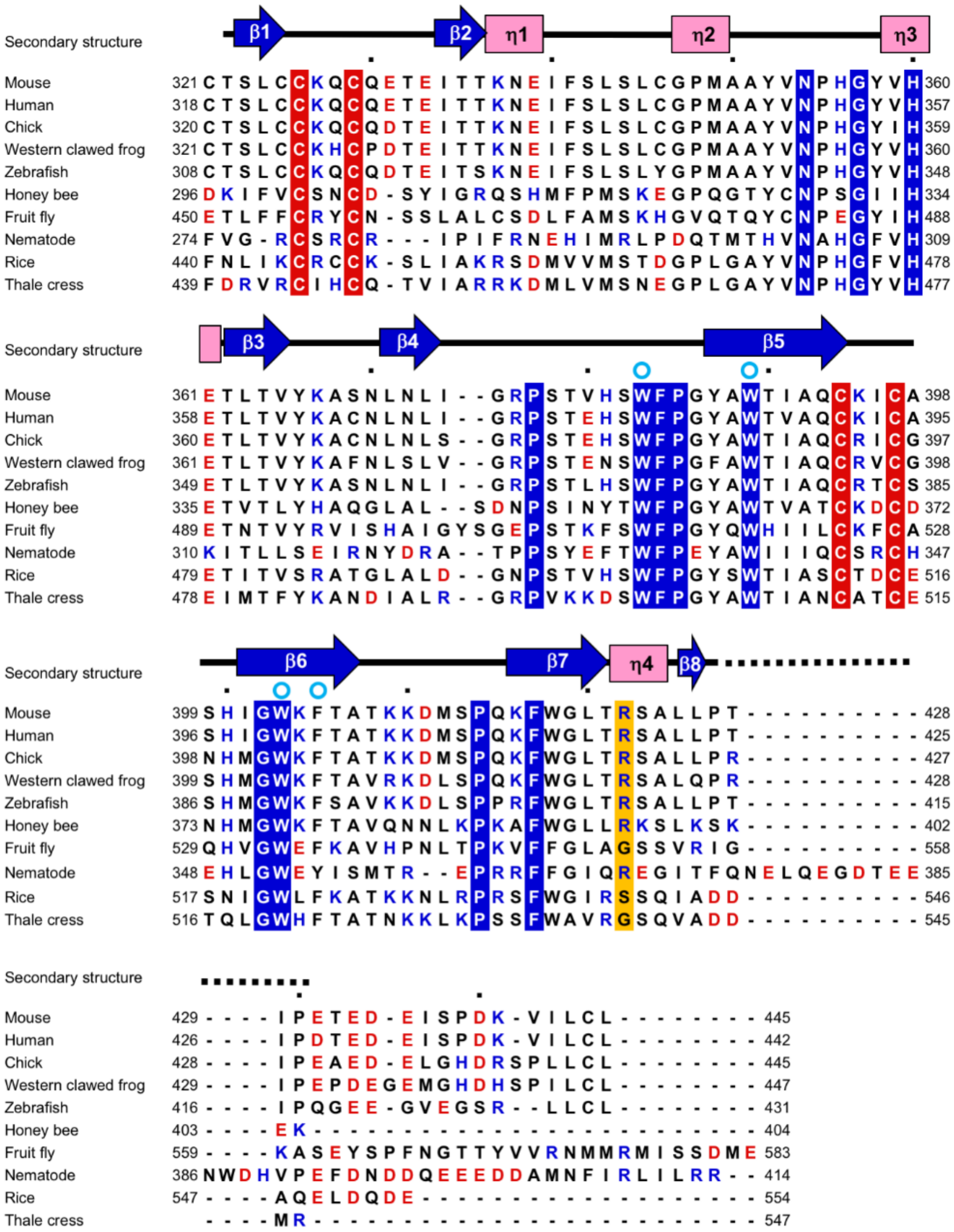

Supplementary Figure 3

Sequence comparison of CRBN TBDs.

Sequence comparison of CRBN TBDs from different sources. The secondary structures of the free form of mouse CRBN TBD, which we previously reported[28], are shown at the top with β-strands (β1-β8; blue arrows), 3_{10}-helices (η1-η4; pink bars), loop (solid lines) and disordered residues (dotted lines). β-strands form anti-parallel β-sheets, the β1-β2-β8 strands and the β4-β5-β6-β7-β3 strands in (Figure 2 b). Three short 3_{10}-helices: (η1,η3 and η4) are flanking the N- or C-terminal ends of the β-strands (β2, β3 and β7, respectively) and the last (η2) is located at the tip of the long flexible β2-β3 loop, which is disordered in the thalidomide-binding TBD (Figure 3 b and c). Acidic (D, E) and basic (K, R, H) residues are designated with red and blue letters, respectively. Residues which form the tri-Trp pocket and directly contact thalidomide are marked with cyan circles. Completely conserved residues are highlighted in blue and zinc-coordinated cysteines of the CXXC motifs in red. Residues involved in the nonsense mutation R419X of human CRBN are highlighted in orange. Mice and humans have only three amino acid differences: mouse Ser369 (loop β3-β4), Val380 (loop β4-β5) and Ile391 (β5) are replaced with Cys366, Glu377 and Val388 in humans, respectively.

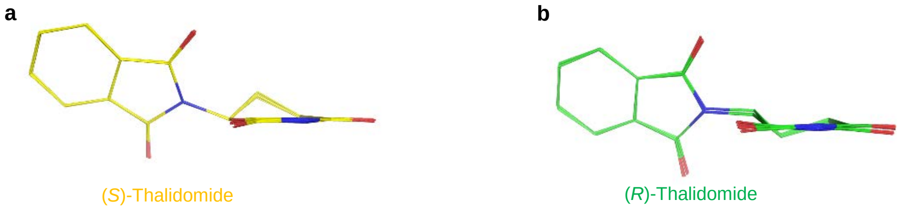

Supplementary Figure 4
Structural comparison of (*S*)- or (*R*)-thalidomides in the asymmetric unit

(**a**) Superpose of 16 molecules of the (*S*)-thalidomides bound to mouse CRBN TBD in the asymmetric unit of the complex crystal. The average of the root-mean-square (rms) deviation is shown as 0.056.
(**b**) As in (a) but for (*R*)-thalidomides. The average of the rms deviation is shown as 0.074.

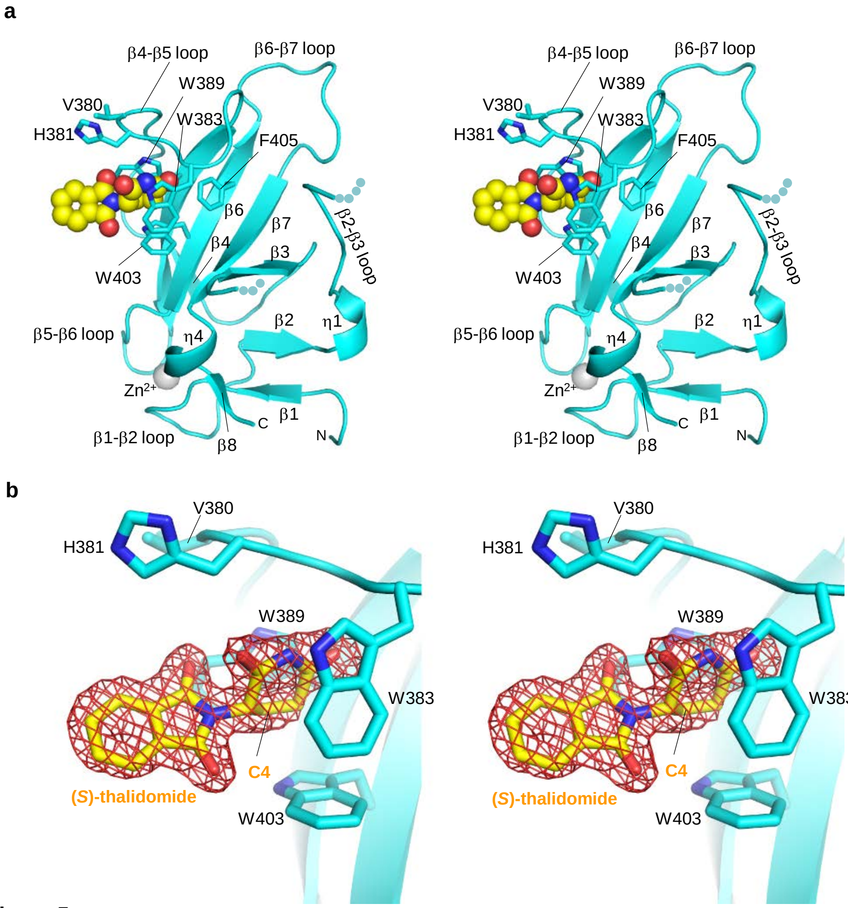

Supplementary Figure 5
Structure of CRBN TBD prepared in the presence of racemic thalidomide.

(**a**) A stereoview of mouse CRBN TBD (cyan) bound to thalidomide (space-filling model; yellow for C, blue for N and red for O) prepared with racemic thalidomide. The glutarimide group of thalidomide is docked into the tri-Trp pocket (three Trp and single Val, His and Phe residues shown as stick models). The bound thalidomide was found to be the (*S*)-enantiomer.
(**b**) A stereoview of the composite omit electron density map for thalidomide bound to tri-Trp pocket in mouse TBD. The map is shown as a red mesh in 1σ contour. The bound thalidomide is the (*S*)-enantiomer (yellow) displaying the C4-*endo* puckered conformation, as observed in the (*S*)-thalidomide-bound form prepared with (*S*)-thalidomide. The crystal structure was solved and refined with protein, ion and water molecules but without thalidomide molecules the map corresponding to the bound thalidomide molecules was calculated. The (*S*)-thalidomide (yellow) molecule, but not (*R*)-thalidomide, fits into the electron density.

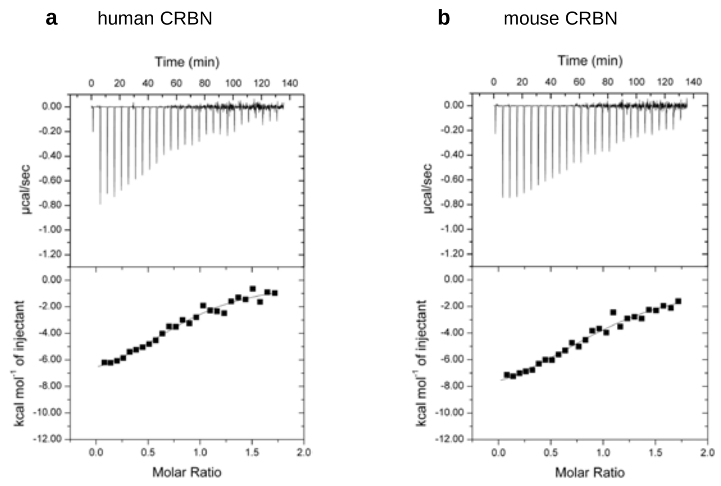

Supplementary Figure 6
Binding assay of glutarimide with TBD of CRBN using isothermal titration calorimetry (ITC).

(**a**) Binding of glutamide to TBD of human CRBN. Reverse titration was performed with injections of 1.5 μl of 1,200 μM TBD into 150 μM glutarimide (250 μl) at 20° C. The injections were performed over a period of 4 sec with a 300-sec interval between injections, and the final concentration reached was 200 μM (26 injections total). TBD exhibited a K_D value of 36.0 ± 7.5 μM, with *ΔH* and *TΔS* values of -8.31 ± 0.6 and +2.4 kcal/mol, respectively.
(**b**) As in *a*, but for binding of glutamide to TBD of mouse CRBN. TBD exhibited a K_D value of 51.3 ± 0.4 μM, with *ΔH* and *TΔS* values of -10.0 ± 0.8 and +4.2 kcal/mol, respectively.

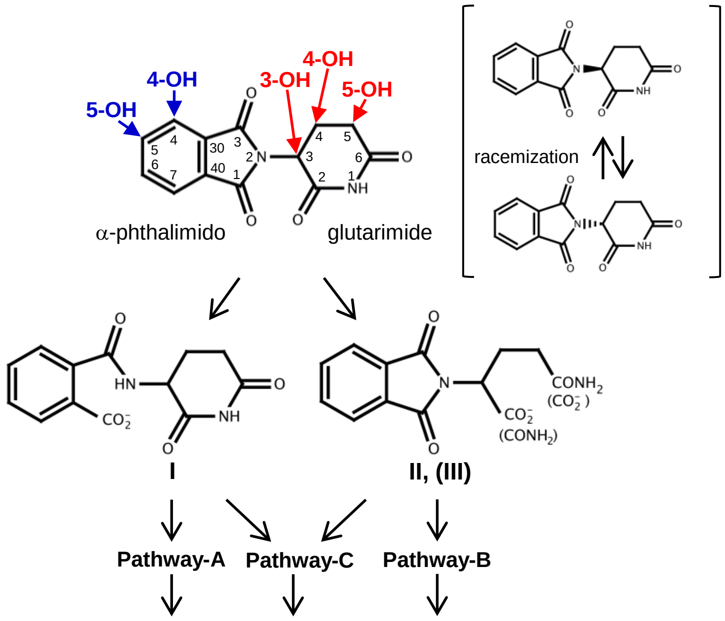

Supplementary Figure 7
Metabolites and hydrolysis of thalidomides.

Summary of thalidomide racemization, main thalidomide metabolites and major hydrolysis pathways. Hydrolysis yields primary products α-(o-carboxybenzamido) glutarimide (I), phthaloylglutamine (II), or phthaloylisolglutamine (III) by ring opening. Compounds in pathway A retain the intact glutarimide ring, while compounds in pathway B retain the phthalimido ring but not the glutarimide ring, and compounds in pathway C have both rings opened.

b

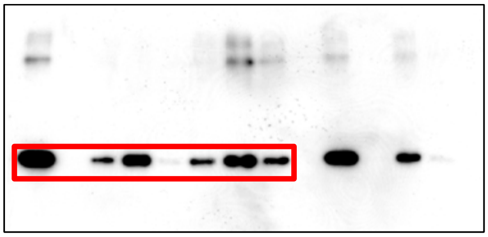

c

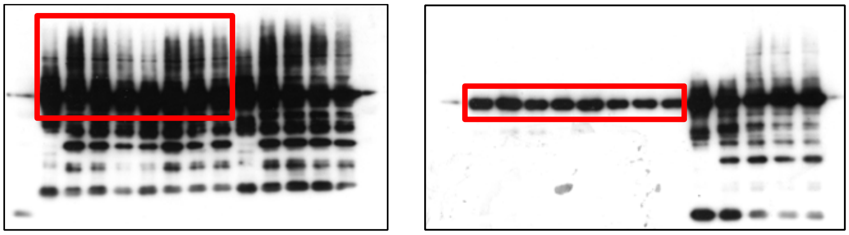

Supplementary Figure 8.
Full-length blots used to generate Figure 1b and c.

a

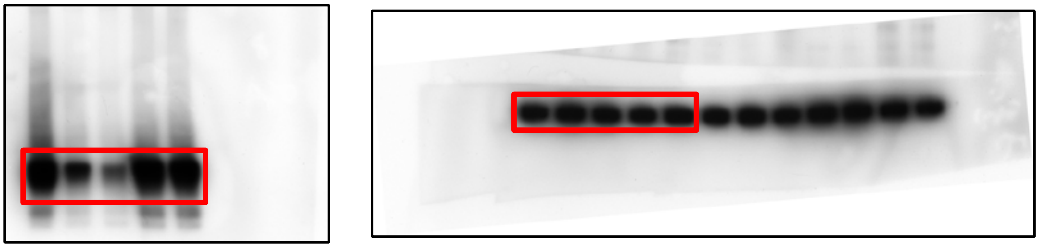

b

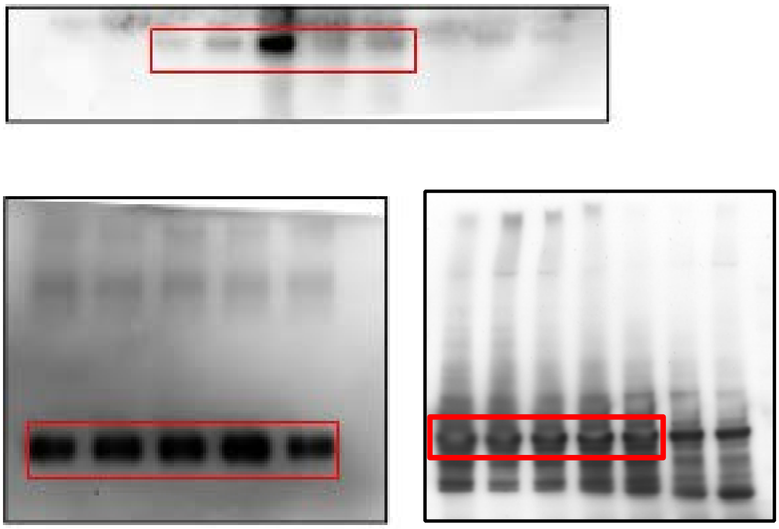

Supplementary Figure 9
Full-length blots used to generate Figure 2a and b.

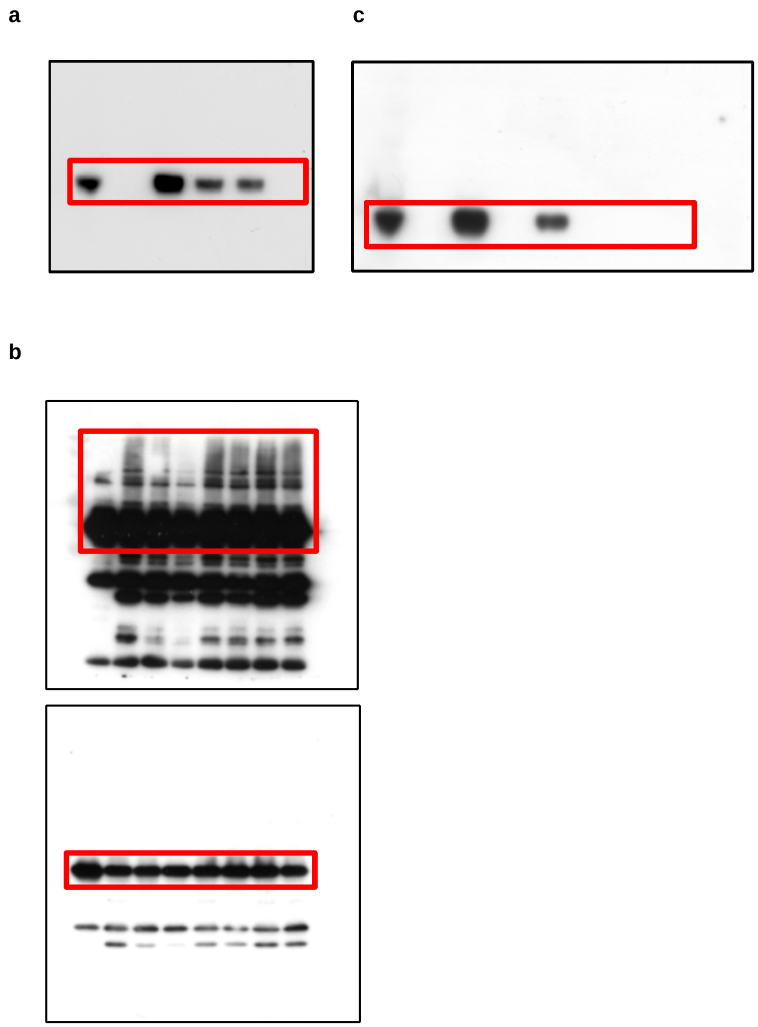

Supplementary Figure 10
Full-length blots used to generate Figure 8a, b and c.
